# Current State-of-Art and New Trends on Lipid Nanoparticles (SLN and NLC) for Oral Drug Delivery

**DOI:** 10.1155/2012/750891

**Published:** 2011-11-24

**Authors:** Patrícia Severino, Tatiana Andreani, Ana Sofia Macedo, Joana F. Fangueiro, Maria Helena A. Santana, Amélia M. Silva, Eliana B. Souto

**Affiliations:** ^1^Department of Biotechnological Processes, School of Chemical Engineering, State University of Campinas (UNICAMP), 13083-970 Campinas, SP, Brazil; ^2^Faculty of Health Sciences, Fernando Pessoa University (FCS-UFP), Rua Carlos da Maia, 296, 4200-150 Porto, Portugal; ^3^Department of Biology and Environment, University of Trás-os Montes e Alto Douro, 5001-801 Vila Real, Portugal; ^4^Centre for Research and Technology of Agro-Environmental and Biological Sciences, 5001-801 Vila Real, Portugal; ^5^Institute of Biotechnology and Bioengineering, Centre of Genomics and Biotechnology, University of Trás-os-Montes and Alto Douro (CGB-UTAD/IBB), P.O. Box 1013, 5001-801 Vila Real, Portugal

## Abstract

Lipids and lipid nanoparticles are extensively employed as oral-delivery systems for drugs and other active ingredients. These have been exploited for many features in the field of pharmaceutical technology. Lipids usually enhance drug absorption in the gastrointestinal tract (GIT), and when formulated as nanoparticles, these molecules improve mucosal adhesion due to small particle size and increasing their GIT residence time. In addition, lipid nanoparticles may also protect the loaded drugs from chemical and enzymatic degradation and gradually release drug molecules from the lipid matrix into blood, resulting in improved therapeutic profiles compared to free drug. Therefore, due to their physiological and biodegradable properties, lipid molecules may decrease adverse side effects and chronic toxicity of the drug-delivery systems when compared to other of polymeric nature. This paper highlights the importance of lipid nanoparticles to modify the release profile and the pharmacokinetic parameters of drugs when administrated through oral route.

## 1. Introduction

The use of lipid particles in pharmaceutical technology has been reported for several years. The first approach of using lipid microparticles was described by Eldem et al. [1], reporting the production by high-speed stirring of a melted lipid phase in a hot surfactant solution obtaining an emulsion. Solid microparticles are formed when this emulsion is cooled to room temperature, and the lipid recrystallizes. The obtained products were called “lipid nanopellets”, and they have been developed for oral administration [2]. Lipospheres were described by Domb applying a sonication process [3–5]. To overcome the drawbacks associated to the traditional colloidal systems [6], such as emulsions [7], liposomes [8], and polymeric nanoparticles [9], solid lipid nanoparticles (SLN) [10, 11] have been developed for similar purposes [12]. 

SLN are biocompatible and biodegradable and have been used for controlled drug delivery and specific targeting. These colloidal carriers consist of a lipid matrix that should be solid at both room and body temperatures, having a mean particle size between 50 nm and 1000 nm [13, 14].

A clear advantage of the use of lipid particles as drug-carrier systems is the fact that the matrix is composed of physiological components, that is, excipients with generally recognized as safe (GRAS) status for oral and topical administration, which decreases the cytotoxicity. SLN have been already tested as site-specific carriers particularly for drugs that have a relatively fast metabolism and are quickly eliminated from the blood, that is, peptides and proteins [15].

The cytotoxicity of SLN can be attributed to nonionic emulsifiers and preservative compounds which are used in the production of these systems [16]. SLN prepared up to concentrations of 2.5% lipid do not exhibit any cytotoxic effects *in vitro* [17]. Even concentrations higher than 10% of lipid have been shown a viability of 80% in culture of human granulocytes [18]. In contrast, some polymeric nanoparticles showed complete cell death at concentrations of 0.5%. In addition, a high loading capacity for a broad range of drugs can be achieved, especially if they have lipophilic properties [12, 19].

Due to their physiological and biodegradable properties, SLN have been tested for several administration routes [20, 21], including the oral [22, 23] and peroral [24, 25] routes. 

SLN can be obtained by exchanging the liquid lipid (oil) of the o/w nanoemulsions by a solid lipid [19]. In general, a solid core offers many advantages in comparison to a liquid core [26]. Emulsions and liposomes usually show lack of protection of encapsulated drugs, and drug release as a burst (emulsions) or noncontrolled (from liposomes). SLN possess a solid lipid matrix identical to polymeric nanoparticles. In addition, SLN are of low cost [27], the excipients and production lines are relatively cheap, and the production costs are not much higher than those established for the production of parenteral emulsions [28].

At the turn of the millennium, modifications of SLN, the so-called nanostructured lipid carriers (NLCs), have been introduced to the literature, and these NLC represent nowadays the second generation of lipid nanoparticles. These carrier systems overcome observed limitations of conventional SLN [29]. The main difference between SLN and NLC is the fact that the concept of these latter is performed by nanostructuring the lipid matrix, in order to increase the drug loading and to prevent its leakage, giving more flexibility for modulation of drug release. This approach is achieved by mixing solid lipids with liquid lipids in NLC instead of highly purified lipids with relatively similar molecules in SLN. This mixture has to be solid at least at 40°C. The result is a less-ordered lipid matrix with many imperfections, which can accommodate a higher amount of drug [11].

## 2. Role of Lipids in Oral Delivery

A limiting factor for *in vivo* performance of poorly water-soluble drugs for oral administration is their resistance of being wetted and dissolved into the fluid in the GIT (apart from potential drug degradation in the gut). Thus, the increase in the dissolution rate of poorly water-soluble drugs is relevant for optimizing bioavailability. Over the last 10 years, poorly water-soluble compounds are formulated in lipid nanoparticles for drug administration [30]. The features of lipid nanoparticles for oral and peroral delivery are related with their adhesive properties. Once adhered to the GIT wall, these particles are able to release the drug exactly where it should be absorbed. In addition, the lipids are known to have absorption-promoting properties not only for lipophilic drugs, such as Vitamin E, repaglinide [22], and puerarin [23].

Hydrophilic drugs can also be incorporated in SLN; nevertheless, the affinity between the drug and the lipid needs to be analysed. Therefore, loading hydrophilic drugs in SLN is a challenge due to the tendency of partitioning the encapsulated molecules in the water during the production process of nanoparticles [31]. Successful examples are zidovudine [31], insulin [32], tretinoin [33], and diminazene [34]. There are even differences in the lipid absorption enhancement depending on the structure of the lipids. For example, medium-chain triglycerides (MCT) lipids are more effective than long-chain triglycerides (LCT) [35]. Basically, the body is taking up the lipid and the solubilized drug at the same time. It can be considered as a kind of “Trojan horse” effect [36, 37].

Oral administration of SLN is possible as aqueous dispersion [38] or alternatively transformed into a traditional dosage forms such as tablets, pellets, capsules, or powders in sachets [25, 39]. For this route, all the lipids and surfactants used in traditional dosage forms can be exploited. In addition, all compounds of GRAS status or accepted GRAS status can be employed as well as from the food industry [40]. Since the stomach acidic environment and high ionic strength favour the particle aggregation, aqueous dispersions of lipid nanoparticles might not be suitable to be administered as dosage form. In addition, the presence of food will also have a high impact on their performance [41]. 

The packing of SLN in a sachet for redispersion in water or juice prior to administration will allow an individual dosing by volume of the reconstituted SLN. For the production of tablets, the aqueous SLN dispersions can be used instead of a granulation fluid in the granulation process. Alternatively, SLN can be transferred to a powder (by spray-drying or lyophilization) and added to the tabletting powder mixture. In both cases, it is beneficial to have a higher solid content to avoid the need of having to remove too much water. For cost reasons, spray drying might be the preferred method for transforming SLN dispersions into powders, with the previous addition of a protectant [42]. 

For the production of pellets, the SLN dispersion can be used as a wetting agent in the extrusion process. SLN powders can also be used for the filling of hard gelatine capsules. Alternatively, SLN can be produced directly in liquid PEG 600 and put into soft gelatine capsules. Advantages of the use of SLN for oral and peroral administration are the possibility of drug protection from hydrolysis, as well as the possible increase of drug bioavailability. Prolonged plasma levels has also been postulated due to a controlled, optimized released [22] in combination with general adhesive properties of small particles [43]. The advantage of colloidal drug carriers described above is that they are generally linked to their size in the submicron range. Therefore, the preservation of particle size of colloidal carrier systems after peroral administration is a crucial point. The gastric environment (ionic strength, low pH) may destabilize the SLN and potentially lead to aggregation. However, it is possible to produce stable SLN dispersions by optimizing the surfactant/mixture for each lipid *in vitro* [44].

The drug release from SLN in the GIT is also dependent on the lipase/colipase activity for the GIT digestion of the lipid matrix. The lipase/colipase complex leads to a degradation of food lipids as a prestep of the absorption. *In vitro* degradation assay based on pancreas lipase/colipase complex have been developed to obtain basic information about the degradation velocity of SLN as a function of lipid and surfactant used in the production process [45, 46].

Lipid nanoparticles show great promise to enhance oral bioavailability of some of the most poorly soluble drugs. The physical/chemical characteristics of lipid particulate systems are highly complex due to the existence of a variety of lipid assembly morphologies, the morphology-dependent solubility of drug, the interconversion of assembly morphology as a function of time and chemical structure, and the simultaneous lipid digestion [47].

## 3. Lipid Nanoparticles as Drug Carriers

Lipid nanoparticles show interesting features concerning therapeutic purposes. Their main characteristic is the fact that they are prepared with physiologically well-tolerated lipids [48]. During the last ten years, different substances have been entrapped into lipid nanoparticles (Table 1), ranging from lipophilic [23, 49] and hydrophilic molecules, including labile compounds, such as proteins and peptides [50]. 

### 3.1. Lipid Materials for Oral Administration

The term lipid is used here in a broader sense and includes triglycerides, partial glycerides, fatty acids, steroids, and waxes. However, it is required that matrix maintains the solid state at room temperature, and for this purpose, the selection of lipids is based on the evaluation of their polymorphic, crystallinity, miscibility, and physicochemical structure [11].  Table 2 shows the main lipids employed for the preparation of lipid nanoparticles.

Furthermore, the use of mono- and diglycerides as lipid matrix composition might increase drug solubility compared to highly pure lipids, such as monoacid triglycerides. Naturally occurring oils and fats comprise mixtures of mono-, di-, and triglycerides, containing fatty acids of varying chain length and degree of unsaturation [25, 86]. The melting point of these lipids increases with the length of the fatty acid chain and decreases with the degree of unsaturation. The chemical nature of the lipid is also important, because lipids which form highly crystalline particles with a perfect lattice (e.g., monoacid triglycerides) lead to drug expulsion during storage time. Physicochemically stable lipid nanoparticles will be obtained only when the right surfactant and adjusted concentration have been employed [25].

### 3.2. Determination of Optimal Hydrophile-Lipophile Balance (HLB) Values for Lipid Nanoparticles Dispersions

Emulsifiers are essential to stabilize lipid nanoparticles dispersions and prevent particle agglomeration [87]. The choice of the ideal surfactant for a particular lipid matrix is based on the surfactant properties such as charge, molecular weight, chemical structure, and respective hydrophile-lipophile balance (HLB). All these properties affects the stability of the emulsion [10]. The HLB of an emulsifier is given by the balance between the size and strength of the hydrophilic and the lipophilic groups. All emulsifiers consist of a molecule that combines both hydrophilic and lipophilic groups. Griffin [88] defined the lipophilic emulsifiers as low HLB values (below 9), and hydrophilic emulsifiers as high HLB values (above 11). Those in the range of 9–11 are intermediate [89].

The HLB system is a useful method to choose the ideal emulsifier or blend of emulsifiers for the system, that is, if its required an oil-in-water (o/w), water-in-oil (w/o) [90], or a double (w/o/w) emulsion. Matching the HLB value of the surfactant with the lipid will provide a suitable *in vitro* performance [91].  Table 3 depicts the mainly surfactants employed in the production of lipid nanoparticles.

 Severino et al. [10] determined the HLB value for stearic acid and stearic acid capric/caprylic triglycerides to reach the best combination of surfactants (trioleate sorbitan and polysorbate 80) to obtain a stable lipid nanoparticles emulsion. The HLB value obtained for stearic acid was 15 and for stearic acid capric/caprylic triglycerides was 13.8. Sorbitan trioleate has an HLB value of 1.8 and polysorbate 80 of 15, when used in the ratio 10 : 90, respectively. The surfactant mixtures prepared with different ratios provided well-defined HLB values. Polysorbate 80 is often used in combination with sorbitan trioleate due to their appropriate compatibility attributed to the similar chemical structure (same hydrocarbon chain length) for the production of stable emulsions. 

## 4. Biopharmaceutic and Pharmacokinetic Aspects 

Pharmacokinetic behaviour of drugs loaded in lipid nanoparticles need to differentiate if the drug is present as the released free form or as the associated form with lipid nanoparticles [106]. However, the poor aqueous solubility of some drugs turns difficult the design of pharmaceutical formulations and leads to variable bioavailability [107]. 

Xie et al. [108] reported a significant increase in the bioavailability and extended the systemic circulation of ofloxacin formulated in SLN, which could be attributed to a large surface area of the particles, improving the dissolution rate and level of ofloxacin in the presence of GIT fluids [109, 110], leading to shorter *T*_max⁡_ and higher peak plasma concentration. In addition, lipid nanoparticles may adhere to the GIT wall or enter the intervillar spaces due to their small particle size, increasing their residence time [111]. Moreover, nanoparticles could protect the drug from chemical and enzymatic degradation and gradually release drug from the lipid matrix into blood, [112] resulting in a several-fold increase mean residence time compared with native drug. Han et al. [113] demonstrated that 5 oral doses of tilmicosin loaded in lipid nanoparticles administered every 10 days provided an equivalent therapeutic benefit to 46 daily doses of oral free drugs. *In vitro* release profile demonstrated that tilmicosin loaded in lipid nanoparticles followed a sustained release profile, and *in vivo* results showed that nanoparticles remained effective for a longer period of time, which was attributed to sustained release of the drug and also to enhanced antibacterial activity by the SLN. 

Pandita et al. [114] developed paclitaxel loaded in SLN with the aim at improving the oral bioavailability of this antineoplastic drug. *In vitro* studies of SLN formulation exhibited an initial low burst effect within 24 h followed by a slow and sustained release. Statistical analysis of *in vivo* experiments concluded that the oral bioavailability of paclitaxel loaded in SLN was significantly higher than the control group. 

Yuan et al. [115] produced stearic acid-SLN with a fluorescence marked for evaluation of *in vivo* pathway by oral administration. About 30% of SLN transport was efficient, where particles were absorbed following linear mechanism in the GIT. The release profile in plasma increased with the increasing of dosage depicting two concentration peaks. The first peak of SLN in blood took place during 1-2 h, attributed to the fast uptake of SLN from the GIT into systematic circulation. Drug concentration began to decrease attributed to the uptake by and the distribution of SLN among particular organs. The second peak occurred at about 6–8 h, and the maximum concentrations were lower than that of the first peak.

## 5. Toxicology

Lipid nanoparticles are well tolerated in living systems, since they are made from physiological compounds leading to the metabolic pathways [22, 28]. For this purpose, studies focusing on nanotoxicology comprise cytotoxicity and genotoxicity analysis [116]. However, such effects often occur first at rather in high concentrations and the subtler effects that arise at lower concentrations, without necessarily causing cell death, also need to be considered. One the most important effect is DNA damage, since an increased genetic instability is associated with cancer development [117]. The interaction with proteins and cells are an essential focus in assessing and understanding compatibility and toxicity. Cell and nanoparticle reactions of interest include cellular uptake and processing of nanoparticle in various routes, effects on cell signalling, membrane perturbations, influence on the cellular electron transfer cascades, production of cytokines, chemokines, and reactive oxygen species (ROS), transcytosis and intercellular transport, gene regulation overt toxic reactivity, no observable toxicity, and cell necrosis or apoptosis. *In vitro* culture of cell lines or primary cells on plastic plates are employed in a wide varieties of assays and reflect the variety of possible physiologic responses to nanoparticles *in vivo* and all possible cell processing routes and natural reactions [118]. 

Silva et al. [119] studied the toxicity of SLN and risperidone loaded SLN with Caco-2 cells by (4,5-dimthylthiazol-2-yl)2,5-diphenyl-tetrazolium bromide (MTT) assay. The results suggest that all formulations evaluated are biocompatible with Caco-2 cells and well tolerated by the GIT. Similar results have been reported elsewhere [120, 121]. This test evaluates the mitochondrial function as a measurement of cell viability, which allows the detection of dead cells before they lose their integrity and shape. The amount of viable cells after SLN exposure was performed by the MTT assay with Caco-2 cell models, which are a well-established *in vitro* model that mimics the intestinal barrier and is often used to assess the permeability and transport of oral drugs [122]. Other authors have also reported that SLN show biocompatibility, which increase their attractiveness for drug-delivery applications [120].

## 6. Marketed Products and Current Studies 

Since early nineties, researchers turned their attention to lipid nanoparticles because of their nontoxicity and cost/effectiveness relationship [12]. In spite of the advantages, formulating with lipid nanoparticles has been suffering some drawbacks. Because of the GIT conditions, most of promising drugs do not reach clinical trials. The stability of particles must be comprehensively tested due to pH changes and ionic strength as well as the drug release upon enzymatic degradation [123]. Lipid nanoparticles absorption through GIT occurs via transcellular (through M cells or enterocytes) or paracellular (diffusion between cells). If the major drug uptake occurs through M cells, the portal vein to the liver is bypassed, resulting in higher drug concentrations to the lymph rather than to plasma [124]. Despite the low number of lipid nanoparticles formulations on the market for drug delivery, Mucosolvan retard capsules (Boehringer-Ingelheim) is a story of success [125]. Mucosolvan retard capsules was the first generation. It was produced by high-speed stirring of a melted lipid phase in a hot surfactant solution obtaining an emulsion. This emulsion was then cooled down to room temperature obtaining the so-called “lipid nanopellets for oral administration” [126]. 

Successful *in vivo* studies also include rifampicin, isoniazid, and pyrazinamide that are used in tuberculosis treatment. These drugs achieved higher bioavailability when incorporated into SLN compared to the free solutions. Rifampicin has poor cellular penetration which requires high doses to reach effective concentrations. Rifamsolin is a rifampicin-loaded SLN under preclinical phase by AlphaRx. The methodology employed for production is acceptable by the regulatory agencies and has been addressed by various papers and patents [127]. 

Poor water-soluble drugs, as camptothecin, vinpocetine, and fenofibrate, can have their solubilization improved if incorporated into SLN [124, 128]. Another example is insulin, commonly administered parenterally in the treatment of diabetes mellitus. Injections are often painful and must be administered daily, which result in low patient compliance [129]. Unfortunately, oral administration of insulin, produced by solvent emulsification-evaporation method based on a w/o/w double emulsion, has limitations such as low bioavailability due to degradation in the stomach, inactivation and degradation by proteolytic enzymes, and low permeability across the intestinal epithelium because of lack of lipophilicity and high molecular weight [124, 129]. The main advantages of incorporate insulin into SLN would be the enhancement of transmucosal transport and protection from the degradation in the GIT.

## 7. Conclusions

Lipids and lipid nanoparticles are promising for oral and peroral administration route for drugs, proteins, and peptides. Theses matrices are able to promoting controlled release of drugs in GIT and reducing absorption variability. In addition, these matrices can be absorption as food lipids together with drugs improving the bioavailability. These systems present several advantages, including drug protection and excipients of GRAS status, which decreases the danger of acute and chronic toxicity. In addition, the oral administration of lipids nanoparticles is possible as aqueous dispersion or alternatively transformed into a traditional dosage forms such as tablets, pellets, capsules, or powders in sachets.

## Figures and Tables

**Table 1:** Examples of drugs, miscellaneous active ingredients and macrocyclic skeletons incorporated into lipid nanoparticles.

Incorporated drug or substance	Lipid	Advantageous	System	References
3′-Azido-3′-deoxythymidine palmitate	Trilaurin	Stable after autoclaving, and can be lyophilized and rehydrated	SLN	[51]

5-Fluorouracil	Dynasa 114 and Dynasan 118	Prolonged release in simulated colonic medium	SLN	[52]

Apomorphine	Glyceryl monostearate, polyethylene glycol monostearate	Enhanced the bioavailability in rats	SLN	[20]

Ascorbyl palmitate	Imwitor 900 and Labrafil M1944	Viscoelastic measurements is appropriate for topical/dermal application	NLC	[53]

Baclofen	Stearic acid	Significantly higher drug concentrations in plasma	SLN	[54]

Benzyl nicotinate	Dynasan 116	Increased oxygenation in the skin	SLN	[55]

Calcitonin	Trimyristin	Improvement of the efficiency of such carriers for oral delivery of proteins	SLN	[56]

Camptothecin	Monostearin and Soybean Oil 788	Stable and high performance delivery system	NLC	[57, 58]

Clozapine	Trimyristin, tripalmitin, and tristearin	Improvement of bioavailability	SLN	[59]

Cyclosporin A	glyceryl monostearate, and glyceryl palmitostearate	Controlled release	SLN	[60, 61]

Dexamethasone	Compritol 888 ATO	Drug delivery topical use	SLN	[62]

Diazepam	Compritol ATO 888 and Imwitor 900 K	Prolonged release	SLN	[63]

Doxorubicin	Glyceryl caprate	Enhanced apoptotic death	SLN	[64]

Gonadotropin release hormone	Monostearin	Prolonged release	SLN	[65]

Hydrocortisone	Monoglyceride, chain length of the fatty acid moiety	SLN stable with release properties	SLN	[66]

Ibuprofen	stearic acid, triluarin, tripalmitin	Stable formulation and negligible cell cytotoxicity	SLN	[67]

Idarubicin	Emulsifying wax	Potential to deliver anticancer drugs	SLN	[68]

Insulin	Stearic acid, octadecyl alcohol, cetyl palmitate, glyceryl monostearate, glyceryl palmitostearate, glyceryl tripalmitate, glyceryl behenate	Promising for oral delivery of proteins	SLN	[50]

Ketoprofen	mixture of beeswax and carnauba wax	SLN with beeswax content exhibited faster drug release as compared carnauba wax	SLN	[69]

Lopinavir	Compritol 888 ATO	Bioavailability enhanced	SLN	[70]

Nimesulide	Glyceryl behenate, palmitostearate, glyceryl tristearate	Sustained drug release	SLN	[71]

Penciclovir	Glyceryl monostearate	Provide a good skin targeting	SLN	[72]

Progesterone	Monostearin, stearic acid and oleic acid	Potential drug delivery system for oral administration	NLC	[73, 74]

Repaglinide	Glycerol monostearate and tristearin	Toxicity study indicated that the SLN were well tolerated	SLN	[22, 49]

Salbutamol sulphate	Monostearin and PEG2000	Formulation accelerate release of hydrophilic small molecule drugs	SLN	[75]

Tetracycline	glyceryl monostearate and stearic acid	Sustained release	SLN	[76]

**Table 2:** Lipids used for lipid nanoparticles production.

Lipids	References
Triglycerides	
Trimyristin (Dynasan 114)	[11]
Tripalmitin (Dynasan 116)	[77]
Tristearin (Dynasan 118)	[11]
Mono, di and triglycerides mixtures	
Witeposol bases	[78]
Glyceryl monostearate (Imwitor 900)	[22]
Glyceryl behenate (Compritol 888 ATO)	[79]
Glyceryl palmitostearate (Precirol ATO 5)	[80]
Waxes	
Beeswax	[81]
Cetyl palmitate	[82]
Hard fats	
Stearic acid	[10]
Palmitic acid	[83]
Behenic acid	[84]
Other lipids	
Miglyol 812	[11]
Paraffin	[85]

**Table 3:** Emulsifiers used for the production of lipid nanoparticles.

Emulsifiers/coemulsifiers	HLB	References
Lecithin	4–9	[92, 93]
Poloxamer 188	29	[94]
Poloxamer 407	21.5	[56, 95]
Tyloxapol	13	[96]
Polysorbate 20	16.7	[92]
Polysorbate 60	14.9	[97]
Polysorbate 80	15	[10, 11]
Sodium cholate	18	[98]
Sodium glycocholate	14.9	[99]
Taurodeoxycholic acid sodium	13-14	[100]
Butanol and Butyric acid	7–9	[101]
Cetylpyridinium chloride	~15	[102]
Sodium dodecyl sulphate	40	[103]
Sodium oleate	18	[99]
Polyvinyl alcohol	15–19	[104]
Cremophor EL	12–14	[105]
